# Description of Material Properties of Degraded and Damaged Segments of Multi-Leaf Masonry in Analyses of Large Three-Dimensional Structures

**DOI:** 10.3390/ma16114076

**Published:** 2023-05-30

**Authors:** Czesław Miedziałowski, Adam Walendziuk

**Affiliations:** Department of Building Structures and Structural Mechanics, Faculty of Civil Engineering and Environmental Sciences, Bialystok University of Technology, Wiejska 45E, 15-351 Białystok, Poland; c.miedzialowski@pb.edu.pl

**Keywords:** masonry, non-homogenous materials, degradation, damage, macro-element

## Abstract

This article focuses on the description of material properties of segments of masonry structures in three-dimensional analyses. It mainly considers degraded and damaged multi-leaf masonry walls. In the beginning, the causes of degradation and damage to masonry are described with examples. It was reported that the analysis of such structures is difficult due to the adequate description of the mechanical properties in the individual segments of the structure and the amount of computational cost of large three-dimensional structures. Next, a method of describing large fragments of masonry structures by means of macro-elements was proposed. The formulation of such macro-elements in three-dimensional and two-dimensional problems was given by introducing limits of variation in material parameters and damage of structures expressed by the limits of integration of macro-elements with specified internal structures. Then, it was stated that such macro-elements can be used to build computational models by the finite element method, which allows the analysis of the deformation–stress state, and at the same time, reduce the number of unknowns in such issues. A strategy for performing analyses and examples of practical applications in masonry analyses were proposed. It was reported that the results of the analyses can be used to plan the repairs and strengthening of structures. Finally, the conducted considerations and proposals were summarised, as well as examples of practical applications.

## 1. Introduction

A significant part of the materials used in the past as well as nowadays in the construction industry are heterogeneous materials and structures such as concrete, reinforced concrete, and masonry. Masonry as a construction material has been used for centuries. Unreinforced masonry can be defined as a composite of two interconnected components—masonry units and mortar. The classification (taxonomy) of the internal arrangements of masonry is extensive with regard to masonry made up of ceramic as well as stone elements and various layers (leaves) [1,2]. In addition to the strength of the components, the influence of an internal structure of the masonry (periodic, quasi-periodic, chaotic textures) on the formation of damage is still being explored. Especially for historical masonry structures, attention is also paid to structural arrangement and the internal structure of masonry walls [3]. Essential interrelated parameters of construction are the durability of component materials and material properties. These parameters are influenced by such factors as wall texture and its construction (single- or multi-leaf), unit shape and size, the volumetric ratio between components, and the mechanical properties of mortar and units—Young’s modulus, Poisson’s ratio, and compressive, tensile, and shear strength. Observations and research studies indicate the variability of these parameters over time [4,5,6,7]. A variety of environmental impacts affect embedded materials and buildings from their establishment.

**Figure 1:**
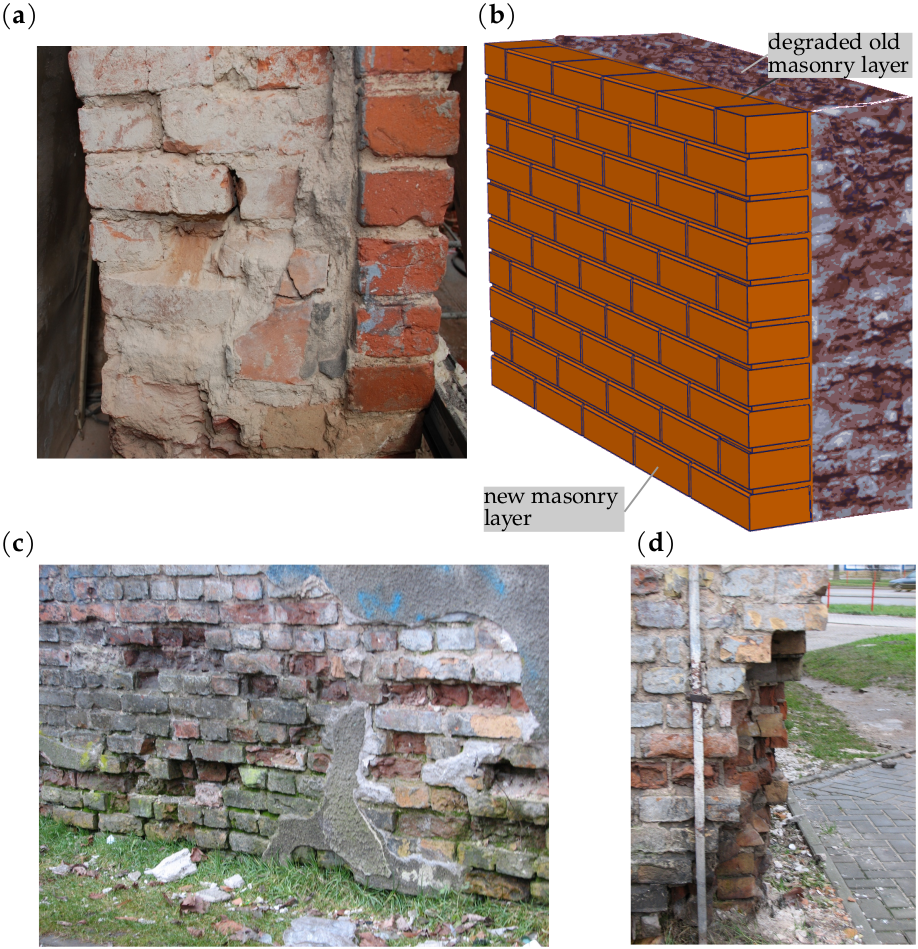
Examples of structures and degradation of masonry fragments (**a**–**d**).

During the realisation and utilisation of structures, physical, chemical, and biological interactions cause processes associated with unfavourable changes in the components, leading to gradual deterioration and degradation of properties of the materials used in structures. Damage due to overloading or frost effects can also occur during a lifetime. It is a natural process difficult to be stopped. A case in point is multi-leaf walls (including degraded ones), in the description of which should be distinguished segments in different states of technical condition. Examples of such degraded masonry structures are shown in Figure 1. Changes in stress states in structures are caused by the degradation of materials, deformations, changes in the purpose (function), and loads of the facility [8]. Degradation and ageing of materials and damages are influenced by the environment, including temperature effects, precipitation, or capillary rising of water from the subsoil. A change in the state of stress in the structure leads to damage in the form of discontinuities and cracks. In some geographical regions, water is an important factor affecting the durability of masonry. Freezing in the pores of the material leads to frost damage (flaking, spalling, or cracking) [9]. It also induces other corrosive processes, changes the texture of the brick, and allows the growth of microorganisms, leading to biodeterioration. Adverse impacts and changes in the properties of materials cause the need to assess the current strength condition and then protect them by increasing their load-bearing capacity and stiffness. Before any action is undertaken, it is important to recognise the scheme of working and stress distribution in a structure composed of masonry units and binders, considering the component arrangement and the mechanisms of damage.

The issues of materials joining are considered in engineering in many fields and aspects [10]. The analysis of heterogeneous materials investigates the interactions of the components and contact zones are also taken into account. These are studied at different scales of observation by assuming homogeneity (using homogenisation) or describing the internal arrangement of components in material. In numerical analyses of such materials, interface-type elements are used to describe the connections [11,12]. These have been used among others in modelling various material structures, for example, masonry [13] and concrete [14]. Material inhomogeneities are also introduced into structural materials by implementing modern repair methods through the injection or bonding of composite or steel materials to reinforced concrete or masonry elements. The scope also includes issues related to the renovation and strengthening of masonry structures and methods based on numerical analysis are useful during their implementation.

For the analysis of masonry structures, many numerical modelling methods have been developed to simulate the behaviour of masonry. In the most general approach, a distinction can be made between continuum models and discrete models. Continuum models are based mainly on the finite element method (FEM) [15]. Discrete models consider the medium as an assembly of distinct bodies interacting along the boundaries (DEM) [16]. A synthetic overview and comparison of the results of these methods can be found in the paper [17]. Depending on the level of accuracy of the description of the medium, the following modelling strategies are distinguished [18]: detailed micro-modelling, simplified micro-modelling, and macro-modelling. In detailed micro-modelling units and mortar, in joints are represented as continuous materials bonded by interface discontinuum elements. Simplified micro-modelling uses the geometric expansion of the units separated by discontinuous elements that simulate the behaviour of the mortar joints and unit-mortar interface. In macro-modelling masonry components: units, mortar, and unit–mortar interface are smeared out in a homogeneous continuum. The aforementioned approaches are based on the representation of the solid medium at the micro-, meso-, and macro-scale. Proposals for more detailed modelling strategy classifications in application to masonry structures are given in papers [19,20]. Among the methods currently being developed, particular attention is paid to multi-scale modelling by analysing the issue at several scales of observation [21], e.g., at the meso- and macro-scale.

Static analysis of masonry structures in spatial (3D) schemes needs the development of large-scale computational models [22]. The analysis of large real spatial structures in multi-year periods of utilisation and calculations in spatial schemes often require the considerable computing power of computers and the use of special computing procedures and techniques [23,24]. One way to solve this problem is through the multi-scale modelling of structures [25,26,27]. In order to reduce the computational cost, this paper proposes the use of multilayer macro-elements that model geometric and mechanical properties of fragments of structures. The proposed formulation of the description of segments (fragments) of the structure with differently localised zones with degradation and damage, as well as the multi-stage analysis, is filling the scientific and research gap. The description of masonry fragments can be regarded as a certain way of numerical homogenisation of masonry in a multi-scale approach [28]. The formulation of such elements and their properties is the main focus of this work. A proposal for a stepwise analysis and examples of the application of such a description to masonry structures will also be presented. Especially in the case of historical masonry walls, we encounter various forms of degradation and ways of implementing repair and strengthening [29,30,31,32]. A reliable description of the mechanism of static work of such masonry structures requires the use of spatial (3D) models, formulated, e.g., in the methodology of the finite element method.

## 2. Problem Formulation

**Figure 2:**
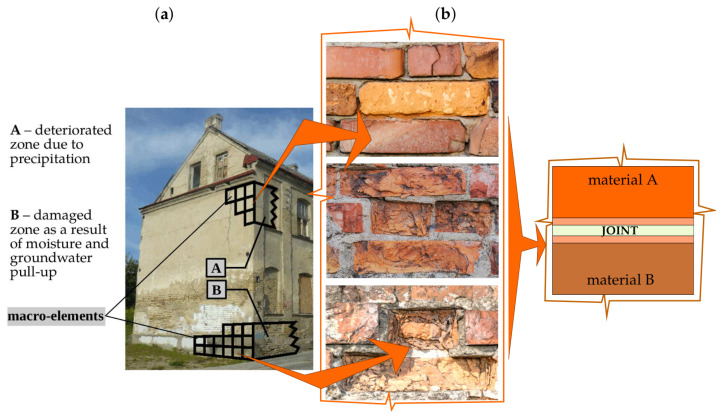
An example of a structure composed of heterogeneous and degraded materials: (**a**) different degradation zones in a building; (**b**) brick arrangement, local degradation forms, and meso-scale model.

In masonry structures, within the range of its technical condition considered, there are fragments in different levels of degradation and damage (Figure 2).

Degradation is understood as a local change in the stiffness of the material, expressed through mass losses, cracking, surface flaking, and, as a consequence, changes in the mechanical parameters E,ν,R. Damage is treated as breaking a continuity of masonry and an exclusion of damaged areas or volumes from static work. The description of degradation and damage is analysed at two scales: at the structural scale, where an equivalent homogeneous medium is considered, and at the meso-scale, where the complex heterogeneous masonry mesostructure is taken into account. The developed method of description consists of separating segments of a certain size in the analysed structural model, referred to as macro-elements. In each macro-element, the existing (actual) state of degradation of the structure and damage in the form of discontinuity is assumed. As a result of integration in the sub-areas, matrices describing the stiffnesses of the macro-elements are determined. The stiffness of the whole structure is described by the set of all macro-elements.

**Figure 3:**
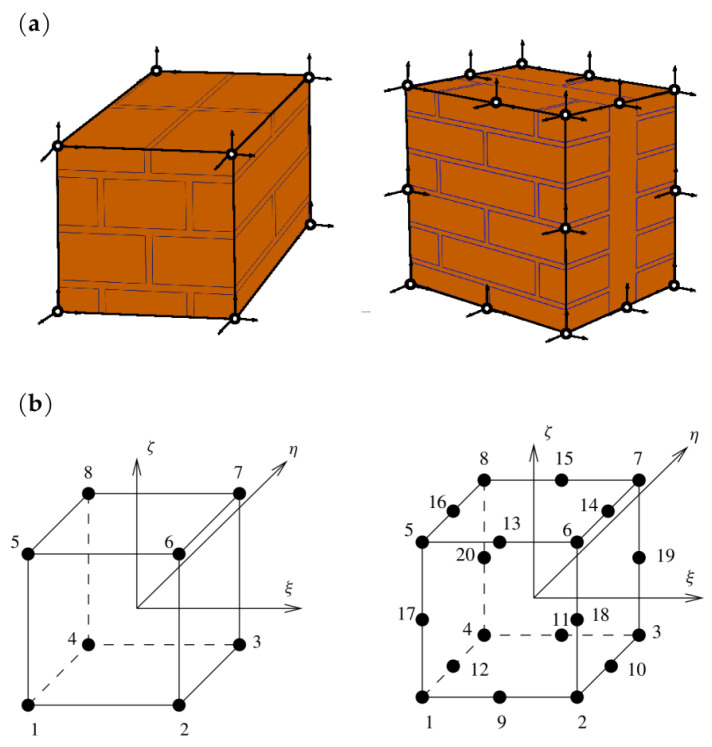
Proposed finite macro-elements describing segments of a structure: (**a**) internal structures of macro-elements in general cases; (**b**) 8- and 20-node finite elements.

A model presented in the work enables us to take into account the variation in physical properties appearing in sub-areas (sub-spaces) of a structure, the damaged fragments of a structure by introducing the limits of variation in material parameters, and the damage of a structure expressed by macro-element integration limits. The model describing larger areas (volumes) is proposed, allowing optimal modelling of the material properties of a structure in a three-dimensional scheme, e.g., by the finite element method, and avoiding high computational costs by using macro-elements and staged analyses. Depending on the dimensions of the analysed structure and the adopted discretisation method—the dimensions of repetitive masonry fragments—it is proposed to use 8- and 20-noded macro-elements, as shown in Figure 3. In this paper, considerations are limited to walls with a regular arrangement of masonry units.

In order to describe the phenomena occurring, areas covering materials with different properties are identified, assuming the continuity of displacements at the boundaries of these areas. The unknowns are located at the nodes and form a global displacement vector q. In this vector, a number of displacements in the zone of the cohesion are selected and an element e is spanned on the nodes. The displacement components of the nodes of this element form a vector $u_{e}$. In the analysis, it is proposed to use macro-elements that sample internal structures, as shown in Figure 3a. The finite macro-elements are spanned on 8 or 20 nodes, and the displacement components of the nodes of these elements have 24 and 60 components, respectively:

(1) $$\begin{array}{cc} u_{e}^{\left(8\right)} & ={\left\{\begin{array}{c} u_{1},\;v_{1},\;w_{1},\;\;\ldots ,\;\;\;u_{8},\;v_{8},\;w_{8} \end{array}\right\}}^{T}, \end{array}$$

(2) $$\begin{array}{cc} u_{e}^{\left(20\right)} & ={\left\{\begin{array}{c} u_{1},\;v_{1},\;w_{1},\;\;\ldots ,\;\;\;u_{20},\;v_{20},\;w_{20} \end{array}\right\}}^{T}. \end{array}$$

The displacement field in heterogeneous media, in which three directions of variation in physical properties of materials are observed, is assumed to be approximated by shape functions $N_{i}^{\left(e\right)}$. In the 8-node element, the polynomials approximating the displacement field are linear:

(3) $$\begin{array}{c} N_{i}^{\left(8\right)}=\frac{1}{8}\left(1+\xi \xi_{i}\right)\left(1+\eta \eta_{i}\right)\left(1+\xi \xi_{i}\right),\;i=1,\ldots ,8 \end{array}$$

whereas in the 20-node element these are second-degree polynomials [33] of the type:

(4) $$\begin{array}{cc} N_{i}^{\left(20\right)} & =\frac{1}{8}\left(1+\xi \xi_{i}\right)\left(1+\eta \eta_{i}\right)\left(1+\zeta \zeta_{i}\right)\left(\xi \xi_{i}+\eta \eta_{i}+\zeta \zeta_{i}-2\right),\;i=1,\ldots ,8 \end{array}$$

(5) $$\begin{array}{cc} N_{i}^{\left(20\right)} & =\frac{1}{4}\left(1-a^{2}\right)\left(1+bb_{i}\right)\left(1+cc_{i}\right),\;a,b,c\in \left\{\eta ,\xi ,\zeta \right\},\;i=9,\ldots ,20 \end{array}$$

where $\xi_{i},\eta_{i},\zeta_{i}$ are the natural coordinates of the node *i*.

The strain energy of the entire structure can be written by the equation:

(6) $$\begin{array}{cc} W_{\epsilon } & =\frac{1}{2}\int_{V}\epsilon^{T}\sigma dV=\frac{1}{2}\int_{V}\epsilon^{T}E\epsilon dV=\frac{1}{2}\int_{V}{\left(\mathrm{Bu}\right)}^{T}E\left(\mathrm{Bu}\right)dV=\\ \; & =\frac{1}{2}q^{T}\sum_{e=1}^{elem}\left[R_{e}^{T}K_{e}R_{e}\right]\;q=\frac{1}{2}q^{T}\mathrm{Kq}\;, \end{array}$$

where:

       E    —elasticity matrix;
       B    —strain matrix;
       $R_{e}$  —an allocation (incidence) matrix, assigns degrees of freedom to elements;
       $K_{e}$  —macro-element stiffness matrix.

**Figure 4:**
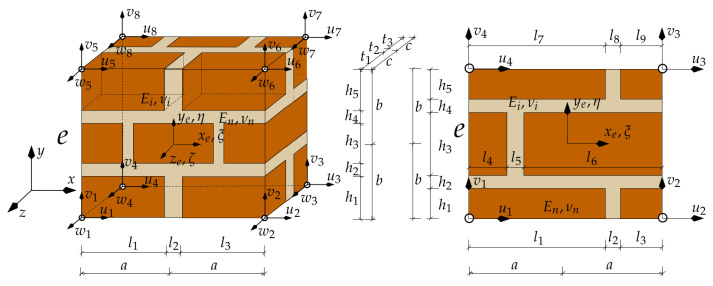
Sample internal structures of macro-elements with layers in three and two directions.

The macro-element stiffness matrix is calculated by integrating the expression for the internal strain energy in sub-spaces (sub-areas). Sample macro-element structures in spatial and flat problems are shown in Figure 4.

In spatial issues:

(7) $$K_{e}=\int_{V_{1}}B^{T}E^{\left(1\right)}B\;dV+\ldots +\int_{V_{j}}B^{T}E^{\left(j\right)}B\;dV+\ldots +\int_{V_{n}}B^{T}E^{\left(n\right)}B\;dV.\;$$

**Figure 5:**
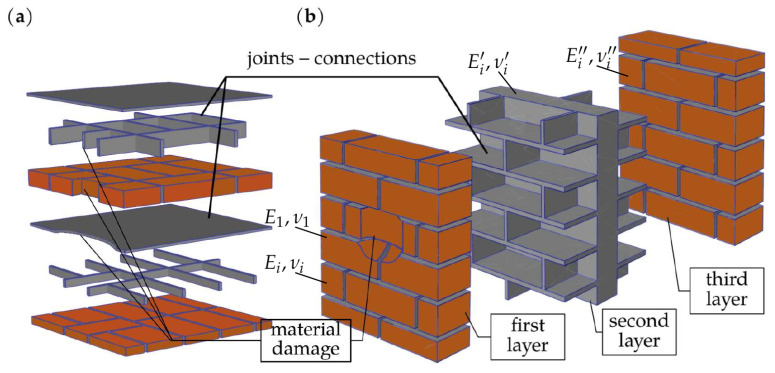
Integration sub-spaces of macro-element with damage: (**a**) repetitive layers (courses) of solid masonry; (**b**) multi-leaf masonry.

Sub-areas of integration are fragments of materials with specific characteristics used in the construction of the wall. Figure 5 shows examples of sub-spaces in a wall with a thickness of two bricks and a wall with a three-leaf structure.

Numerical integration is carried out according to the relationship written in the form:

(8) $$\begin{array}{c} K_{e}=\int_{-a}^{-a+l_{1}}dx\int_{-b}^{-b+h_{1}}dy\int_{-c}^{-c+t_{1}}B^{T}E^{\left(1\right)}B\;dz\;+\\ \;+\int_{-a+l_{1}}^{-a+l_{1}+l_{2}}dx\int_{-b}^{-b+h_{1}}dy\int_{-c}^{-c+t_{1}}B^{T}E^{\left(2\right)}B\;dz\;+\;\\ \;\ldots +\int_{-a+\sum l_{i-1}}^{-a+\sum l_{i}}dx\int_{-b+\sum h_{i-1}}^{-b+\sum h_{i}}dy\int_{-c+\sum t_{i-1}}^{-c+\sum t_{i}}B^{T}E^{\left(i\right)}B\;dz\;+\;\\ \;\ldots \;\;+\int_{a-\sum l_{n-1}}^{a}dx\int_{b-\sum h_{i-1}}^{b}dy\int_{c-\sum t_{n-1}}^{c}B^{T}E^{\left(n\right)}B\;dz\; \end{array}$$

in which the symbols l,h,t are shown in Figure 4. The general form of the deformation matrix B (the derivatives of the shape function) is constant in each sub-area:

(9) $$B^{}=\left[\begin{array}{ccccc} B_{1}^{\left(e\right)}, & \ldots , & B_{i}^{\left(e\right)}, & \ldots , & B_{n}^{\left(e\right)} \end{array}\right],$$

(10) $$B_{i}^{\left(e\right)}=LN_{i}^{\left(e\right)}=L\left[\begin{array}{ccc} N_{i}^{\left(e\right)} & 0 & 0\\ 0 & N_{i}^{\left(e\right)} & 0\\ 0 & 0 & N_{i}^{\left(e\right)} \end{array}\right],$$

        *i*         —node number (i=1…n),
        *n*        —number of nodes of the macro-element,
        **L**        —operator matrix,
        $N_{i}^{\left(e\right)}$  —shape functions.

The elasticity matrix in sub-space *j* (bricks, joints), in general varying over time (T), is written in the form:

(11) $$\begin{array}{c} E_{j}^{\left(T\right)}\;\;=\;E_{{}^{j}}\;\;=\;\frac{E_{j}}{\left(1+\nu_{j}\right)\left(1-2\nu_{j}\right)}\left[\begin{array}{cccccc} \left(1-\nu_{{}_{j}}\right) & \nu_{{}_{j}} & \nu_{{}_{j}} & 0 & 0 & 0\\ \nu_{{}_{j}} & \;(1-\nu_{{}_{j}}) & \nu_{{}_{j}} & 0 & 0 & 0\\ \nu_{{}_{j}} & \nu_{{}_{j}} & \;(1-\nu_{{}_{j}}) & 0 & 0 & 0\\ 0 & 0 & 0 & \;\;\frac{\left(1-2\nu_{j}\right)}{2} & 0 & 0\\ 0 & 0 & 0 & 0 & \;\;\frac{\left(1-2\nu_{j}\right)}{2} & 0\\ 0 & 0 & 0 & 0 & 0 & \;\;\frac{\left(1-2\nu_{j}\right)}{2} \end{array}\right]\;. \end{array}$$

**Figure 6:**
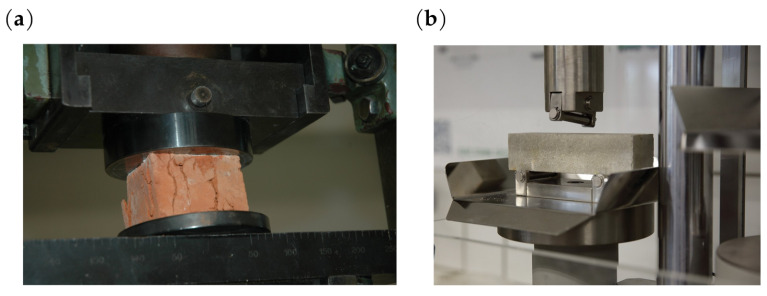
Laboratory testing of materials: (**a**) bricks; (**b**) mortar.

In the notation of matrix E, the superscript (T) is used to emphasise the variability of the parameters over time, but for the sake of clarity, it will be omitted in most notations, as in the notations of other matrices and vectors: K,q,Q. Elements of the matrix E dependent on the material constants, Young’s modulus *E*, and Poisson’s ratio ν of the brick and mortar are generally variable in the adopted sub-spaces *j* and during long-term service life of the structure, and should be determined in laboratory tests [34] (Figure 6).

**Figure 7:**
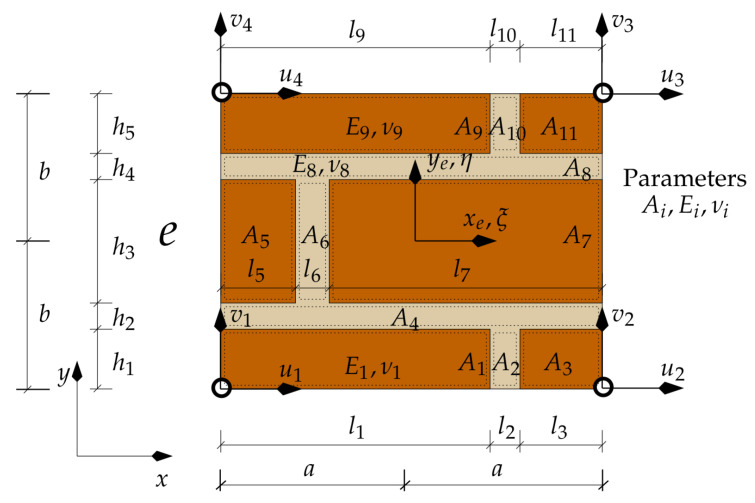
A sample internal structure of thickness-constant macro-element.

Flat models can be used in some cases. In a 2D area composed of sub-areas (Figure 7), integration in sub-area *j*, under certain assumptions, can be carried out analytically, obtaining the explicit form of the macro-element stiffness matrix. The following results are then obtained:

(12) $$\begin{array}{c} \begin{array}{c} K_{e}^{\left(T\right)}\;=\; \end{array}K_{e}=t\int_{-a}^{-a+l_{1}}dx\int_{-b}^{-b+h_{1}}B^{T}E^{\left(1\right)}B\;dy\;+\\ \;+t\int_{-a+l_{1}}^{a-l_{3}}dx\int_{-b}^{-b+h_{1}}B^{T}E^{\left(2\right)}B\;dy\;+\ldots \\ \;\ldots +t\int_{-a+l_{9}}^{a-l_{11}}dx\int_{b-h_{5}}^{b}B^{T}E^{\left(10\right)}B\;dy+t\int_{a-l_{11}}^{a}dx\int_{b-h_{5}}^{b}B^{T}E^{\left(11\right)}B\;dy\;,\; \end{array}$$

and *t* denotes the layer thickness.

Below is enclosed a piece of the code for calculating the first element of the stiffness matrix obtained by integration of expression (12). The selected terms of the sum are equal: 
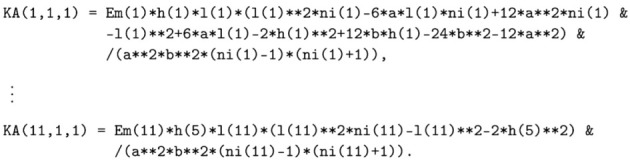


The value of the element (1,1) of the stiffness matrix is then calculated from the formula: 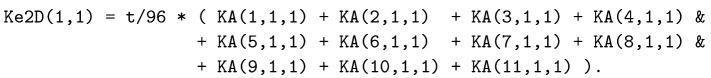


## 3. Strategy for Performance of Analyses

It is proposed that the calculations should be carried out according to a staged algorithm that uses the formulation of the equilibrium equations in the form:

(13) $$K_{i}^{\begin{array}{c} \left(T\right) \end{array}}q_{i}^{\begin{array}{c} \left(T\right) \end{array}}=Q_{i}^{\begin{array}{c} \left(T\right) \end{array}}\;,$$

where:

       $K_{i}^{\begin{array}{c} \left(T\right) \end{array}}$  is variable over time stiffness matrix of the modelled medium;
       $q_{i}^{\begin{array}{c} \left(T\right) \end{array}}\;$  is the vector of unknown node displacements of the model at step *i* of the iteration;
       $Q_{i}^{\begin{array}{c} \left(T\right) \end{array}}$  is the load vector.

**Figure 8:**
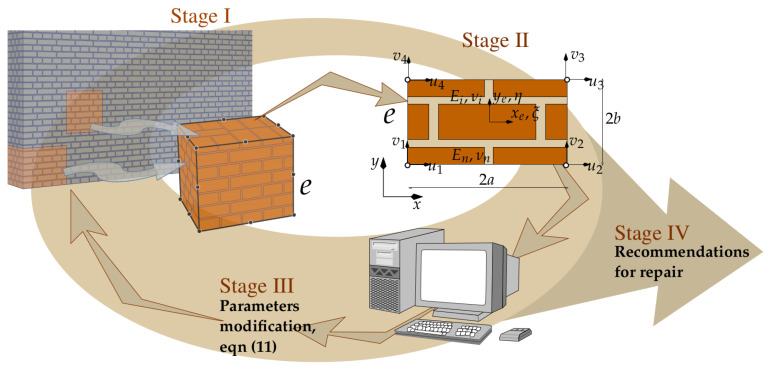
Stages of numerical analysis of heterogeneous materials embedded into a construction.

The formulation makes it possible to model material degradation and damage in segments of large existing structures or to investigate the effects of loading history over years of service on the degradation of masonry and the state of stress level in a structure. The computer implementation of the algorithm takes advantage of the secant stiffness matrix, seeking equilibrium of the system at each load increment. The scheme for carrying out the calculations has been divided into stages, as illustrated in Figure 8.

**Stage** **I**    —analysis of large areas or volumes and macro-element formation;
**Stage** **II**   —analysis of displacements, assessment of stress and strain fields inside the macro-elements;
**Stage** **III**  —parameters modification in a model according to (11) and recomputation;
**Stage** **IV** —recommendations for repairs and strengthening.

**Figure 9:**
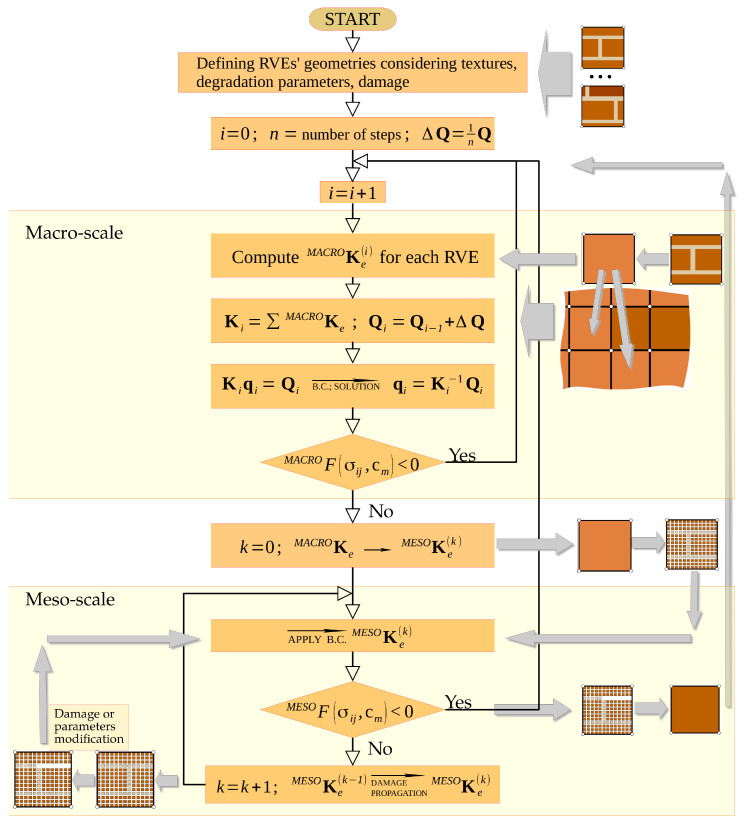
Flowchart of step-by-step computational process.

As stated above, the method of analysis developed consists of separating segments (fragments) of a certain size in the structural model to be analysed, taking into account the unit arrangement and degradation. The unit arrangement (texture) does not need to be regular or repeatable; however, this affects the cost of the computations. For each segment, a specified (existing) state of degradation of the internal structure and the properties of the materials that compose it is assumed, in addition to possible damages and discontinuities of the structure. As a result of the integration in the sub-spaces (sub-areas) of the segments, the matrices used to model the stiffness of the segments (macro-element stiffness matrices) are calculated. The set of all such macro-elements forms the stiffness matrix of the modelled structure $K_{i}$ in successive steps of load increments $Q_{i}$. The load increment can also be interpreted as a time-dependent parameter. Once the displacement field of the model nodes has been determined, the stress state in each sub-area is verified. When the stresses exceed the permissible values, the current damage configuration of the internal structure is determined in each macro-element by searching for an equilibrium configuration at the meso-scale (at the RVE level). For the modified RVE structure, the macro-element stiffness matrix is calculated for the next loading step. The implementation of this calculation procedure is presented in a simplified flowchart (Figure 9), where only the most essential steps of the computational process are indicated. The flowchart is directly related to the numerical analysis strategy presented in Figure 8. It also contains graphical information consistent with the approach presented in Section 2 of this article describing degradation and damage processes. The masonry wall structures analysed are treated as brittle and a simple approach was used in the computational process that does not directly take into account other complex aspects, including that of plasticity. Exceeding the permissible stresses in brittle structures results in the elimination of the area of the structure where damage propagation is identified. This is expressed in a change in the integration limits or modification of the properties of the fragment of the internal structure of the macro-element, as illustrated in Figure 9. The proposed approach employs therefore an isotropic damage model of the internal structure of the macro-element.

In order to perform stress assessment, determine damage zones in heterogeneous materials and structures, as well as to identify damage propagation and degradation mechanisms during the loading process, failure criteria are used [35]. They are formulated analytically in the form of functions of stresses, strains, or their invariants using the results of laboratory strength tests. The safe state of the stress field $\sigma_{ij}$ is defined by the inequality:

(14) $$F(\sigma_{ij},c_{m})<0,$$

and $c_{m}$ denotes the material parameters obtained in laboratory tests. The failure initiation of non-symmetric materials in the range of tensile stresses is verified by the Rankine criterion. The failure of brittle materials also depends on the value of the mean stress. Among the numerous failure criteria used in masonry analyses, the Mohr–Coulomb criterion is often applied [36,37]. In the present work, the criterion is adopted in the form:

(15) $$\frac{1}{2\cos\varphi }\left(\sigma_{i}-\sigma_{j}\right)+\frac{1}{2}\left(\sigma_{i}+\sigma_{j}\right)\tan\varphi <R_{s},\;i,j=1,2,3$$

where φ is the angle of internal friction.

The principal stresses $\sigma_{i}$, $\sigma_{j}$ are determined in macro-element structures from the computed displacements of its nodes. Additionally, the criterion is used to control the state of stress level in joints in which the physical plane of stress is determined, and it is written by an inequality:

(16) $$\left|\tau \right|=c-\sigma_{n}\tan\varphi <R_{s},$$

in which $R_{s}$ is the shear strength of the joint.

## 4. Application Examples

**Figure 10:**
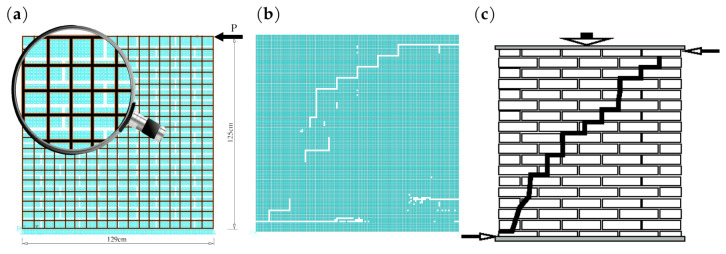
Shear wall: (**a**) model discretisation; (**b**) damage propagation pattern according to the adopted algorithm; (**c**) a form of typical failure mechanism in masonry under shear and compression.

The applicability of the model is demonstrated by examples. They were performed according to the presented step-by-step algorithm for successive load increments in order to reproduce the results of laboratory tests in which degradation over a longer period of time is unimportant. The first example presents an analysis of a fragment of masonry shearing parallel to the load-bearing joints with the geometry given in Figure 10. The discretisation with 324 macro-elements resulted in the division is shown in Figure 10a. The comparative discretisation by standard plane stress elements contains 16,125 finite elements in the size of 1 cm × 1 cm. The macro-elements used have dimensions several times larger, with side lengths in the range of 6–10 cm presented with lines in black. Material parameters were assumed to be the same in the brick and mortar areas, respectively, (degradation and damage of the initial masonry structure were not taken into account). The number of unknowns in the developed models was 722 using macro-elements and 5942 using conventional modelling, respectively. Subsequent calculation steps were carried out according to the algorithm developed and shown in the flowchart. As a result of the solution, the damage distribution in the masonry sample was obtained. An exceedance of the stress values according to the adopted failure criterion was interpreted as material damage, as illustrated in Figure 10b, and compared with a typical form of damage (Figure 10c). Typical forms of damage obtained in laboratory tests of this type of masonry specimens can be found in papers [38,39], among others.

The qualitative similarity of the obtained results of the two presented tests and a consistent trend in the form of damage to the masonry sample are observed. In both presented examples of numerical analyses, the same values of parameters characterising ceramic bricks and mortar were used: Young’s modulus, Poisson’s ratio, compressive strength, tensile strength, and shear strength were equal, respectively, to:

$$\begin{array}{cccc} E^{\left(brick\right)} & =17,500\;\mathrm{MPa}, & E^{\left(mortar\right)} & =2900\;\mathrm{MPa},\\ \nu^{\left(brick\right)} & =0.20,\; & \nu^{\left(mortar\right)} & =0.20,\\ R_{c}^{\left(brick\right)} & =27.1\;\mathrm{MPa}, & R_{c}^{\left(mortar\right)} & =10.0\;\mathrm{MPa},\\ R_{t}^{\left(brick\right)} & =\frac{1}{7}R_{c}^{\left(brick\right)}, & R_{t}^{\left(mortar\right)} & =\frac{1}{5}R_{c}^{\left(mortar\right)},\\ R_{s}^{\left(brick\right)} & =\frac{4}{5}R_{t}^{\left(brick\right)}, & R_{s}^{\left(mortar\right)} & =\frac{4}{5}R_{t}^{\left(mortar\right)}. \end{array}$$

**Figure 11:**
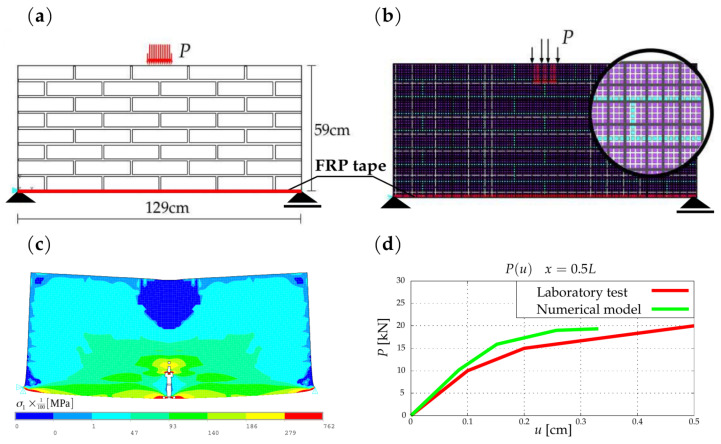
The model analysed: (**a**) scheme and geometry; (**b**) discretisation of the masonry structure; (**c**) the stresses in the masonry; (**d**) the P(u) force–displacement relationship diagram.

A further example refers to a part of brick masonry representing a flexural member of a structure strengthened with an FRP strip placed under the lower course of bricks. The arrangement of the units in the brickwork element studied and the macro-element meshing discretising the masonry units and mortar are shown in Figure 11. A total of 490 macro-elements with side dimensions of 3–5 cm and 7611 elements of the classical FEM discretisation were used. The number of unknowns in the model where macro-elements were used was 1080, compared to 7280 unknown displacements in the classical model. In the numerical experiment, a linear model of the strengthening material was adopted with a value of Young’s modulus $E^{FRP}$ = 240 GPa. The FRP strip material was modelled with bar finite elements, assuming no damage between the strengthening layer and the bricks. The numerical experiment was carried out according to the adopted algorithm in the flowchart. The presented damage form, similarly as in the first example, was determined on the basis of the adopted failure criterion.

The realisation of the real element test is described in the work [40]. The results of the strength laboratory experiments and numerical simulation are shown in Figure 11c,d. The displacement–force relationship graphs obtained in both experiments show convergence. Analysing the differences in the obtained correlations for the two experiments, these were determined to be 16.6% for 0.1 cm displacements, 11.5% for 0.2 cm displacements, and 8.2% for 0.3 cm displacement values. Comparisons of the results show a convergent trend in both experiments.

## 5. Conclusions

The work presents proposals for the description and analysis of heterogeneous structures, composed of dissimilar materials in different states of technical condition and level of degradation. Examples include masonry structures of historical buildings and other masonry structures. The demonstrated method of modelling with macro-elements allows the analysis of large fragments of structures, taking into account their heterogeneity and degradation over time. Stiffness matrices of the macro-element describing such fragments were obtained by numerical or explicit (analytical) integration within the limits of variation in material parameters, degradation, and damage to the structure. This method can be regarded as a kind of numerical homogenisation of the structural arrangement of masonry structures. In this work, such elements were formulated and application examples were given, along with a proposal for staged analysis of structures. The results obtained in the examples show the correct modelling of structures using the proposed method and macro-elements, show the correct tendency of relationships, and quite good convergence of computational and experimental results varying within 8–16%. The modelling method and strategy make it possible to analyse structures in spatial (3D) or flat (2D) schemes and reduce the number of unknowns by about seven times. In engineering practice, there is a need for the repair and rehabilitation of heterogeneous and degraded structures. The modelling approach given in the work allows the assessment of the stress state in structural elements in order to perform the repairs and strengthening of multi-material structures, as illustrated in Figure 11.

## Data Availability

Data sharing is not applicable to this article.
